# Marinated Sea Bream Fillets Enriched with *Lactiplantibacillus plantarum* and *Bifidobacterium animalis* subsp. *lactis*: Brine Optimization and Product Design

**DOI:** 10.3390/foods10030661

**Published:** 2021-03-19

**Authors:** Barbara Speranza, Antonio Bevilacqua, Angela Racioppo, Daniela Campaniello, Milena Sinigaglia, Maria Rosaria Corbo

**Affiliations:** Department of Agriculture, Food, Natural Resources and Engineering, University of Foggia, 71122 Foggia, Italy; barbara.speranza@unifg.it (B.S.); antonio.bevilacqua@unifg.it (A.B.); angela.racioppo@unifg.it (A.R.); daniela.campaniello@unifg.it (D.C.); milena.sinigaglia@unifg.it (M.S.)

**Keywords:** brine, marination, functional strains, optimization, sea bream

## Abstract

This study aimed to design marinated sea bream fillets, inoculated with either *Lactiplantibacillus plantarum* (strains 11, 68, 69) or *Bifidobacterium animalis* subsp. *lactis* DSM 10140. In the first step, the optimization of brine composition was performed through a centroid; the factors of the design were citric acid, vinegar, and salt. As a result of optimization, the optimal composition of brine was set to 0.75% citric acid, 55% vinegar, and 3% NaCl. In the second step, sea bream fillets were inoculated with *L. plantarum* strain 69 and *B. animalis* subsp. *lactis*, marinated and then packed in a conditioning solution (oil or diluted brine); the samples were stored at 4 °C for 21 days. The viability of the strains and sensory scores were assessed. The bacteria retained a high viability throughout storage (21 days); however, the sensory scores were at their highest level for 4 days. In particular, sensory assessment suggested a preference for a conditioning solution with oil, rather than with a diluted brine. In addition, a slightly higher preference was found for *B. animalis* subsp. *lactis*.

## 1. Introduction

The awareness that probiotic foods can have beneficial effects on health has led to an increase of consumer requests for these products. In order for probiotic bacteria to have a beneficial effect on human health, they must survive and remain viable during the expected shelf life of the probiotic food or beverage, without producing negative effects on the sensory properties of the food itself [1].

Results from numerous investigations indicate that probiotics supplied with food are more easily adaptable to the conditions in the gastrointestinal tract and are more effective in maintaining good gut health than probiotics embedded in pharmaceutical preparations [2].

Functional and probiotic foods are mostly enclosed in dairy foods (yogurt, kefir, acidophilus milk, etc.). However, the increase in lactose intolerance and allergies to milk proteins are the leading factors for non-dairy probiotic foods, such as probiotic fermented cereals, fruits, vegetables, and fruit juices [3,4,5,6]. Some studies have focused attention on fermented probiotic seasoned dried sausages [7], fermented soy milk, oat-based cereal bars, olives, and artichokes [8].

Although this trend has affected several segments of the agri-food industry, the fish products sector has not yet been significantly affected by this new generation of food products.

It is well known that fish is widely perceived by consumers as healthy food, because it is a source of polyunsaturated fatty acids, essential minerals and vitamins, and has a high protein content [9,10]. In particular, fish is rich in omega-3 polyunsaturated fatty acids (PUFAs), including docosahexaenoic acid (DHA) and eicosapentaenoic acid (EPA), both well known to prevent cardiovascular diseases [11,12]. In addition, some researchers showed a positive effect on metabolic syndrome and prevention of cardiovascular disease [13,14], as well as on life expectancy and reduction of the incidence of obesity [15].

Some authors proposed the addition of probiotic strains in fermented fish [16,17] and in marinated fish products. In 2012, Speranza et al. [18] proposed an innovative probiotic food—that is, marinated anchovies enriched with *L. plantarum* c19. In another study, marinated swordfish fillets were inoculated with *Lacticaseibacillus paracasei* IMPC 2.1 [19]. The results showed that inoculation successfully ensured the growth of the probiotic strain and controlled the growth of other lactic acid bacteria (LAB).

This technique appears to be an effective means of maintaining the microbial quality of fish fillets and an alternative tool for delivering probiotics. Therefore, the aim of this research was to develop probiotic marinated sea bream fillets. The study was divided into two different steps: 1. brine optimization to ensure the survival of probiotic microorganisms; 2. functional fish product optimization packaged in different solutions.

## 2. Materials and Methods

### 2.1. Microorganisms

Three autochthonous strains of *Lactiplantibacillus plantarum* (previously known as *Lactobacillus plantarum* [20]), namely, strains 11, 68, and 69 isolated from intestinal microbiota of sea bream and with functional properties [21], and *Bifidobacterium animalis* subsp. *lactis* DSM 10140 (Deutsche Sammlung von Mikroorganismen und Zellkulturen) were used throughout this research. The microorganisms were stored at −20 °C in MRS broth (Oxoid, Milan, Italy) added with 33% of sterile glycerol and grown under anaerobic conditions in MRS broth (37 °C for 24 h).

### 2.2. Brine Optimization

#### 2.2.1. Brine Optimization: Experimental Design

Brine optimization was obtained using a simplex centroid. In this research, three independent variables were chosen: vinegar, NaCl, and citric acid, and were included in the range 40–70%, 0–12%, and 0–1.5%, respectively. Each variable was set at 4 different levels, identified with the codes 0 (minimum), 1 (maximum), 0.5 (half point of the interval), and 0.33 (1/3 of the maximum). Table 1 reports the 7 combinations of the centroid and the control (i.e., a further combination where the vinegar, NaCl, and citric acid were set to 0.0%, simulating the optimal conditions). In particular, four centroids were performed for each target strain.

#### 2.2.2. Sampling

Agar diskettes (2% *w*/*v*) were used to simulate a fish fillets model system. Before use, the four probiotic strains were grown in MRS broth (Oxoid) and incubated at 37 °C for 24 h. The microorganisms were centrifuged at 4000 × *g* for 10 min, the supernatant was discarded, and the pellet was suspended in sterile saline solution (0.9% NaCl). This cell suspension (9 log CFU/mL) was used to inoculate a test tube containing a sterile and liquid agar solution (2%, 55 °C) at 8 log CFU/mL. Immediately after inoculation, the agar solution was put in Petri dishes and left to dry for 1 h; then brine solutions were added on the surface of each plate (ratio agar/solution, 1:1.5), and the viable count of probiotics was evaluated after 24 and 48 h. Agar pieces (20 g) were taken and diluted in 180 mL of saline solution in a Stomacher bag (Seward, London, England), then homogenized for 1 min in a Seward Stomacher Lab Blender 400. Serial dilutions of the homogenates were inoculated on MRS agar and incubated at 37 °C for 24–48 h under anaerobic conditions. All tests were performed in duplicate over two different batches.

All microbiological data were modelled using the equation:

(1) $$\Delta \log\;N\;=\;N_{c}\;-\;N_{S}$$

where *N_c_* and *N_s_* are cell numbers (log CFU/g) in the control and in each of the combinations of centroid, thus attaining two sets of data (i.e., Δlog*N*24 and Δlog*N*48), corresponding to the reduction of cell counts observed after 24 and 48 h.

These data were used as inputs for a modelling approach through DoE theory (software Statistica for Windows) to build polynomial equations:

(2) $$y\;=\;\sum_{i=1}^{3}\beta_{i}x_{i}\;+\;\sum_{i\leq }^{3}\sum_{j}^{3}\beta_{ij}x_{i}x_{j}\;+\;\sum_{i\leq }^{3}\sum_{j\leq }^{3}\sum_{k}^{3}\beta_{ijk}x_{i}x_{j}x_{k}\;+\varepsilon$$

where *β_i_*, *β_ij_*, and *β_ijk_* are the coefficients of the individual (*x_i_*) and interactive effects (*x_i_x_j_−x_i_x_j_x_k_*) of the independent variables (vinegar, NaCl, and citric acid); *ε* is the standard error of the model.

Through polynomial equations, prediction profiles were built; in particular, two profiles were evaluated:

(a) Salt (vinegar and citric acid were fixed to the coded level 0, that is, 40% and 0%);
(b) Citric acid (vinegar and salt at 40% and 0%, respectively).

### 2.3. Product Optimization

#### 2.3.1. Sample Preparation

Sea bream of the Adriatic Sea (*Sparus aurata*), supplied by the company Ittica di Dio (Molfetta, Italy) were used. The sea bream specimens were deheaded, gutted, washed, and filleted. The fillets were stripped of skin and immersed in a marinating saline solution consisting of 55% vinegar, 3% NaCl, and 0.75% citric acid for 48 h at 4 °C. Then, the fillets were removed from the solution, inoculated with either *L. plantarum* 69 or *B. animalis* (8 log CFU/g) and packed in high-barrier nylon/polyethylene bags (102 μm) by means of S100-Tecnovac equipment (Tecnovac, San Paolo D’Argon, Bergamo, Italy). Two types of conditioning brines were tested: (1) sample in sunflower oil (O); (2) sample in diluted brine (5.5% vinegar, 0.3% salt, 0.075% citric acid) (S).

During storage at 4 °C for 21 days, samples were periodically analysed as described in the following. All analyses were conducted twice.

#### 2.3.2. Microbiological Analyses

For microbiological analyses, the following media were used: plate count agar (PCA) incubated at 5 °C for 1 week and at 30 °C for 48 h under aerobic conditions for psychrotrophic bacteria and total bacteria count, respectively; *Pseudomonas* agar base (PAB), supplemented with *Pseudomonas* CFC supplement, incubated at 25 °C for 48 h for *Pseudomonadaceae*; violet red bile glucose agar (VRBGA) incubated at 37 °C for 24 h for Enterobacteriaceae; triple sugar iron agar (TSIA) incubated at 37 °C for 24 h for specific spoilage microorganisms (SSOs). All the media and the supplements were from Oxoid (Milan, Italy). The media and the conditions used for probiotic bacteria were: MRS agar for *L. plantarum* 69 (MRSA) and NPNL-MRS agar (NPNL-MRSA) for *Bifidobacterium animalis* subsp. *lactis* 10140, incubated at 37 °C for 48 h. The NPNL solution consisted of nalidixic acid (750 mg/L, Sigma-Aldrich, Milan), paromomycin sulphate (10 mg/L, Sigma-Aldrich), neomycin sulphate (5 mg/L, Sigma-Aldrich), and lithium chloride (150 mg/L, Sigma-Aldrich).

The measurement of pH was conducted in duplicate for each sampling on the fish homogenate with a Crison pH meter micro pH model 2001 (Crison, Barcelona, Spain).

#### 2.3.3. Sensory Analyses

For sensory analyses, the samples were evaluated to assess the sensory scores in terms of colour, texture, odour, and overall acceptability through a quantitative descriptive analysis. The sensory evaluation panel consisted of 20 trained panellists aged between 25 and 50 years, who were students and researchers of the Department of Agriculture, Food, Natural Resources and Engineering (University of Foggia). Initial recruitment of panellists was performed using a questionnaire and pre-selecting members by means of a triangular test for salty and acidic tastes. Before the training, two pre-observation sessions were conducted where 2 persons observed the marinated fillets every day, recording the quality changes from the first day until it was spoiled. In this way, a primary grade system was established, in which 5 was the highest score, indicating the best quality of fish fillets on the first day, and 0 was the lowest score, indicating the spoiled fillets. The training session included 5 meetings during which panellists examined different fish samples to define the evaluation techniques and to become familiar with the organoleptic attributes of marinated fish products. In particular, in the first three sessions, panellists evaluated marinated fillets with a known storage time in order to choose appropriate parameters and modify the score range, making the system more efficient. Then, in sessions four and five, the marinated fillets were assigned three-digit random number blinding codes and evaluated for sensory characteristics. At the end of the session, the panellists were informed of the storage time. Thus, all parameters and the specific range of the score were fixed, completing the scheme used in the test sessions (Table 2).

During the test sessions, marinated fish samples in plastic trays covered with a lid were presented individually to each panellist in random order; in addition to the marinated samples inoculated with probiotics and packed in sunflower oil or in the diluted brine, a master control sample (no probiotic addition) was also presented to panellists. Sensory evaluation was conducted in individual booths under controlled conditions of light (white light), temperature (20 ± 2 °C), and humidity (70% to 85%). The score was assigned using a scale ranging from 0 to 5, as detailed in Table 2. Sample overall quality was an average of three sensory attributes (colour, odour, and texture).

Sensorial data were analysed through a MANOVA (multivariate analysis of variance); time, strain, and sample (SO and SS) were used as categorical predictors. Tukey’s test was used as the post-hoc comparison test (*p* < 0.05).

In addition, spider profiles at selected time intervals were done; mean values were used as input data.

## 3. Results

### 3.1. Brine Optimization

This research intended to develop a probiotic marinated sea bream carpaccio for two reasons: 1. sea bream is one of the most appreciated and consumed fish species at the national level; 2. marinated fish products are very popular with consumers, especially the traditional carpaccio products (cold marinated fish with salt, fruit juices, and acetic and citric acids). Furthermore, the use of mild technologies (generally used for their stabilization) could guarantee the survival of the probiotic microorganism during the processing and storage of the product [18].

During the marinating process, the preservative effect is achieved by an acidification of the product, obtained using solutions based in different amounts of vinegar or acetic acid and salt [22].

In this paper, the optimization of brine was addressed by the use of a mixture design, where each compound (NaCl, vinegar (V), and citric acid (CA)) is a component of a blend. The results after 24 h were analysed as viability loss (VL), whereas modelling was not performed after 48 h because the strains were always below the detection limit.

VL of *L. plantarum* 69 was influenced by the positive and linear terms of NaCl, vinegar, and citric acid and by the interactions vinegar*NaCl and NaCl*citric acid; that is, VL increased when NaCl, vinegar, and citric acid increased. The polynomial equation reads as follows:

*VL* = 0.779[*V*] + 6.818[*NaCl*] + 3.748[*CA*] + 13.480[*V*][*NaCl*] + 7.576[*NaCl*][*CA*]     (R^2^, 0.982) (3)

**Figure 1:**
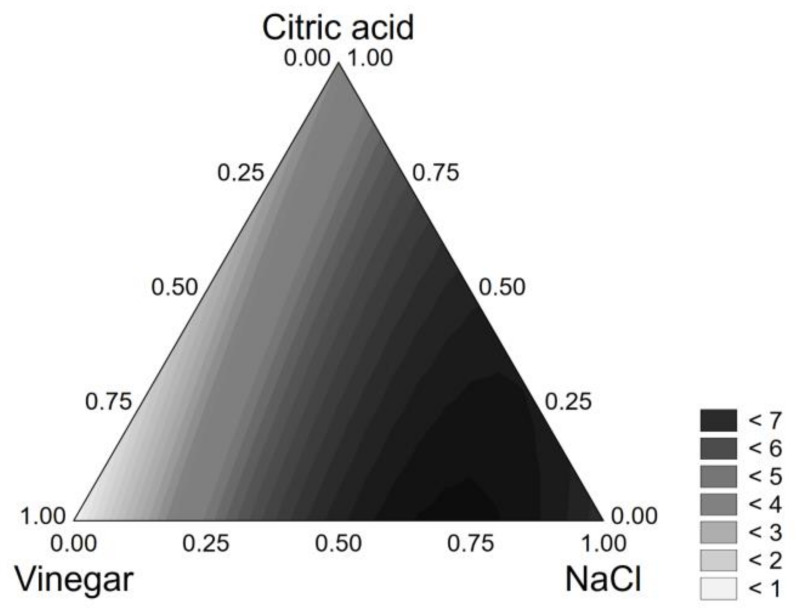
Ternary plot for the viability loss of *L. plantarum* 69 after 24 h in the model system.

The quantitative results are in the ternary plot (Figure 1). Although citric acid and vinegar were significant, the only compound able to exert a quantitative effect was NaCl. In fact, viability loss was maximum (7 log CFU/g) for a code level of salt of 0.75–1.0 (that is, 9–12%). These results confirm the technological robustness of the strains recovered in laboratory media (i.e., inhibition in the presence of 10–12.5% NaCl) [17].

The same modelling approach was used for the other strains. For *L. plantarum* 68, NaCl was significant as an individual term and in interaction with the vinegar; in this case, the least significant variable was vinegar. The polynomial equation reads as follows:

*VL* = 1.414[*V*] + 2.998[*NaCl*] + 6.264[*CA*] + 9.706[*V*][*NaCl*] + 8.080[*NaCl*][*CA*]     (R^2^, 0.922) (4)

**Figure 2:**
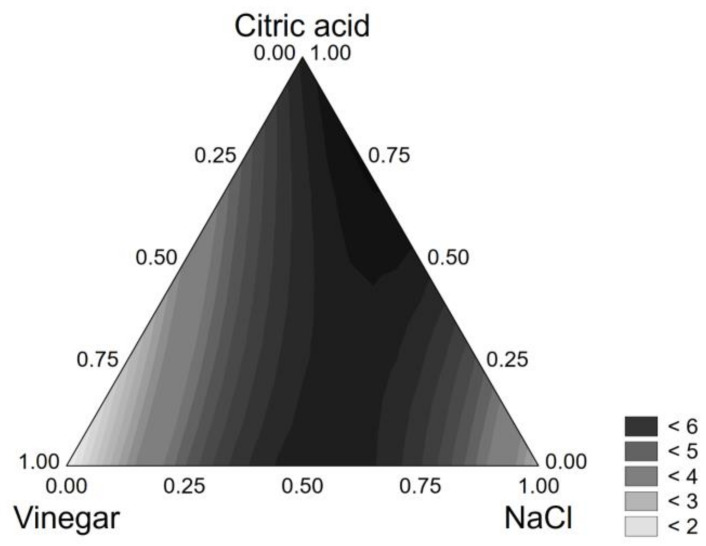
Ternary plot for the viability loss of *L. plantarum* 68 after 24 h in the model system.

For this strain, the highest viability loss (6 log CFU/g) was found for the combination NaCl + citric acid, both at the coded level 0.5 (6% NaCl + 0.75% citric acid) (Figure 2).

*L. plantarum* strain 11 was affected by NaCl and citric acid, both as individual and interactive terms, as well as by vinegar, although its statistical effect was low:

*VL* = 2.040[*V*] + 6.202[*NaCl*] + 6.201[*CA*] + 7.696[*V*][*NaCl*] + 7.683[*NaCl*][*CA*]     (R^2^, 0.988) (5)

**Figure 3:**
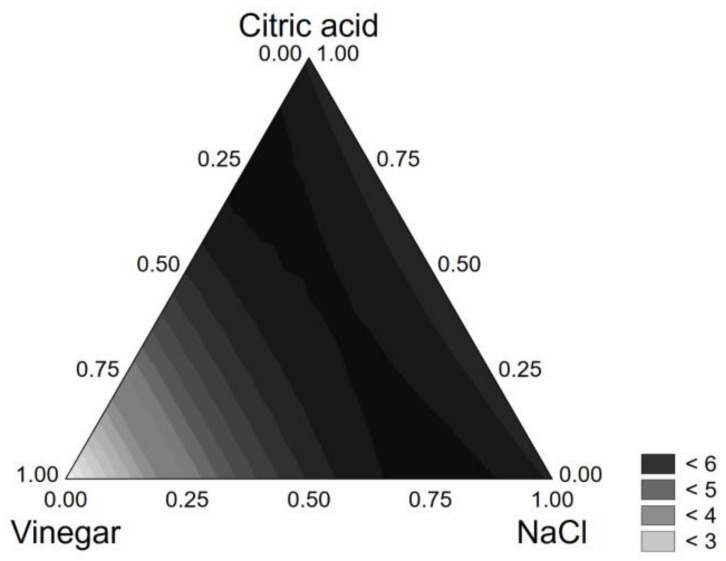
Ternary plot for the viability loss of *L. plantarum* 11 after 24 h in the model system.

This strain experienced a strong viability loss (6 log CFU/g) in many combinations of the design (among others NaCl + citric acid, 0.5 + 0.5; citric acid + vinegar, 0.5 + 0.5; NaCl + vinegar, 0.5 + 0.5; NaCl + citric acid + vinegar, 0.33 + 0.33 + 0.33) (Figure 3); therefore, it was regarded as unsuitable for product design.

Finally, *B. animalis* subsp. *lactis* was influenced by the NaCl and citric acid concentrations (as individual terms) and by the interactions vinegar*citric acid and citric acid*NaCl, as shown in the following equation:

*VL* = 6.397[*NaCl*] + 4.502[*CA*] + 14.220[*V*][*NaCl*] + 5.653[*NaCl*][*CA*]     (R^2^, 0.973) (6)

**Figure 4:**
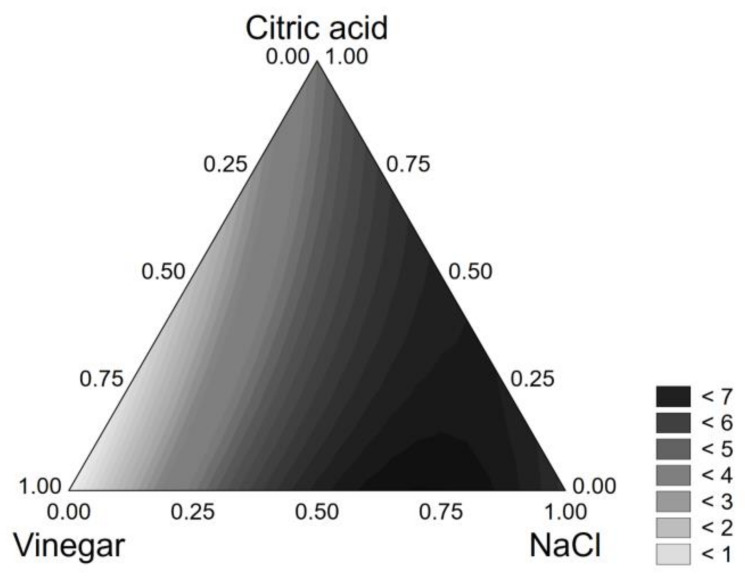
Ternary plot for the viability loss of *B. animalis* subsp. *lactis* DSM 10140 after 24 h in the model system.

The highest VL was found for NaCl amounts of 9–12% (coded levels 0.75–1.0) (Figure 4).

**Figure 5:**
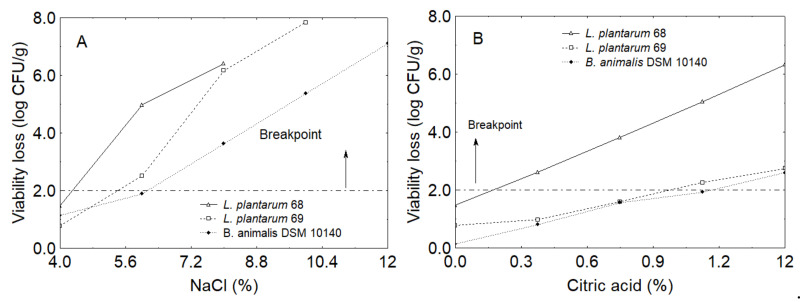
Prediction profiles: individual effect of NaCl (**A**) and citric acid (**B**) on the viability loss of *B. animalis* subsp. *lactis* DSM 10140, *L. plantarum* 68, and *L. plantarum* 69 in the model system (agar dishes).

Ternary plots show the quantitative effect of the three factors of centroid; however, they do not allow an extrapolation of the individual effect of each independent variable. Thus, prediction profiles for salt and citric acid were built (Figure 5). Generally, viability loss increased when salt and citric acid increased (e.g., up to 7 log CFU/g for *B. animalis* at 12% NaCl or 6 log CFU/g with 1.5% citric acid).

For optimization, an arbitrary breakpoint was set (2 log CFU/g) and acceptable levels of salt and citric acid were recovered from prediction profiles, because it was not possible to find a saddle point (i.e., a combination of variables resulting in the lowest viability loss). Considering that the initial inoculum was 8 log CFU/g, a decrease of ca. 2 log CFU/g could be considered acceptable, as the minimum level required for a probiotic strain in a food or probiotic drink is 6 log CFU/g [22]. Table 3 shows the predicted coded values for these variables for each strain. *L. plantarum* 68 was cut off because it experienced a strong viability loss and the predicted coded values were too low for a marinating solution (NaCl at 0.04% and citric acid at 0.17%). The critical values of NaCl and citric acid were set at 3% and 0.75%, as shown by the prediction for the *B. animalis*. Vinegar was not a limiting factor and could also be set at maximum level (code level 1, equal to 70%). However, the choice of the composition of brine should also be based on other elements, such as the effect on the organoleptic characteristics of products; therefore, the vinegar concentration was set to 55%. Thus, the final composition chosen for the brine was 55% vinegar, 3% NaCl, 0.75% citric acid.

Only salt and citric acid had significant effects on the viability of the tested strains. Acids and salt are generally used to delay the action of bacteria and enzymes in fish. Ray and Bhunia [23] demonstrated that the addition of 0.3% acetic acid had a bactericidal effect, mainly against Gram-negative bacteria. Moreover, the interaction of salt with acids ensures not only specific product characteristics such as taste, appearance, or texture, but also environmental conditions that delay the growth of microorganisms that cause spoilage [24]. As reported by other authors [17,25], the technological robustness and the resistance to salt and acids are strain-dependent; strains 11 and 68 showed a poor technological aptitude, thus, they were excluded from the second step of this research.

### 3.2. Product Optimization

Salt resistance and growth in acid conditions can be considered the necessary characteristics for a strain to be used in marinated fish products. Furthermore, important features that probiotic strains should fulfil are viability and the ability to create pleasant flavours, prolong shelf life, and have a useful impact on consumer health [26].

The aim of the second step was to assess these traits in real conditions by focusing on microbiological data, sensory scores, and pH.

As a result of conditioning in the brine optimized in Section 3.1, the pH of the fish fillets decreased from 6.3 to 3.75–3.80, whereas specific spoilage microorganisms (SSOs), *Pseudomonas* spp., enterobacteria, and lactic acid bacteria were below the detection limit.

Table 4 reports the cell counts of probiotics on fish packed in sunflower oil (O) or in the diluted brine (S) during 3 weeks of refrigerated storage. Neither *L. plantarum* 69 nor *B. animalis* experienced significant viability loss over time, while psychrotrophic bacteria, *Pseudomonas* spp., enterobacteria, and SSOs were below the detection limit (data not shown). The pH was at 3.7–3.8 for 21 days. As expected, microbial activity was inhibited due to the combined effect of saline and acid solutions; it is well known that the marinating process provides food safety with microbial inactivation [27].

Several studies reported that fish could be considered a suitable matrix supporting the viability of LAB, mainly probiotic strains [19,28]. Giribaldi et al. [19] reported that the probiotic strain *L. paracasei* IMPC 2.1, inoculated in marinated ready-to-eat swordfish fillets, was not only able to survive up to 90 days of refrigerated storage, but also to improve the fatty acid profile and oxidative stability of the studied products.

**Figure 6:**
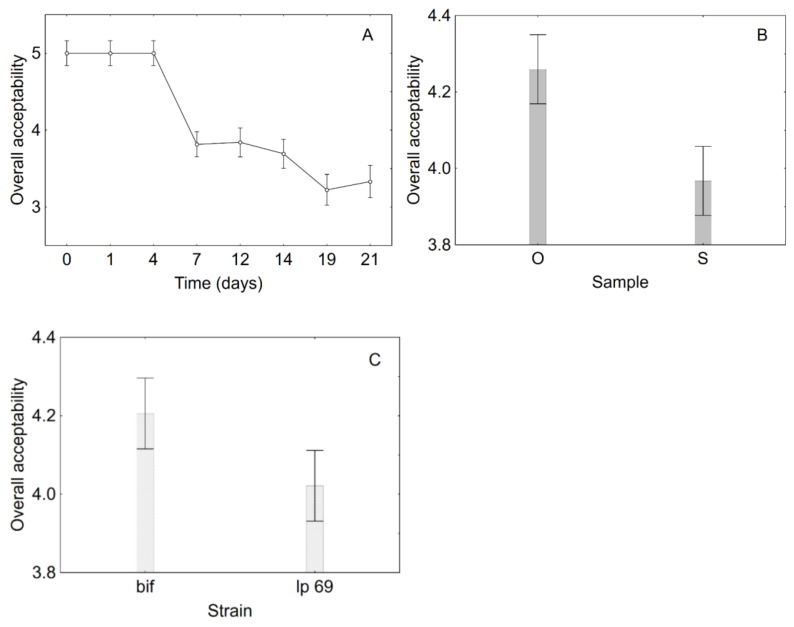
Decomposition of the statistical hypothesis for the effects of time (**A**), sample (**B**), and strain (**C**) on the overall acceptability. O, products in sunflower oil; S, products in diluted brine. Bif, *B. animalis* subsp. *lactis* DSM 10140; lp 69, *L. plantarum* 69.

In this study, the cell loads of *L. plantarum* 69 and *B. animalis* 10140 never reached the critical value during the 21 days of the experiment. Thus, a preliminary evaluation of the shelf life was determined based on the sensory scores. Generally, time was the most significant factor for colour, odour, texture, and overall acceptability; the kind of sample (packaged in oil or in diluted brine) also played a role. Figure 6 shows the decomposition of the statistical hypothesis for the overall quality. As expected, sensory scores decreased over time (Figure 6A; *p* < 0.001), with a higher decrease in diluted brine (Figure 6B; *p* < 0.001) and with *L. plantarum* 69 (Figure 6C; *p*, 0.004), although the critical breakpoint for the sensory scores was never attained.

**Figure 7:**
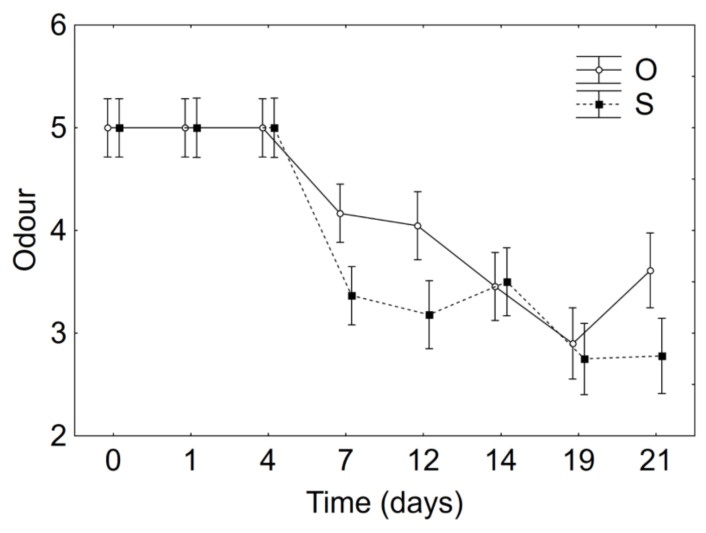
Decomposition of the statistical hypothesis for the interaction sample*time on odour. O, products in sunflower oil; S, products in diluted brine.

The time*sample interaction was important for the odour, because the samples in oil retained higher scores up to 4 days (Figure 7; *p*, 0.001).

**Figure 8:**
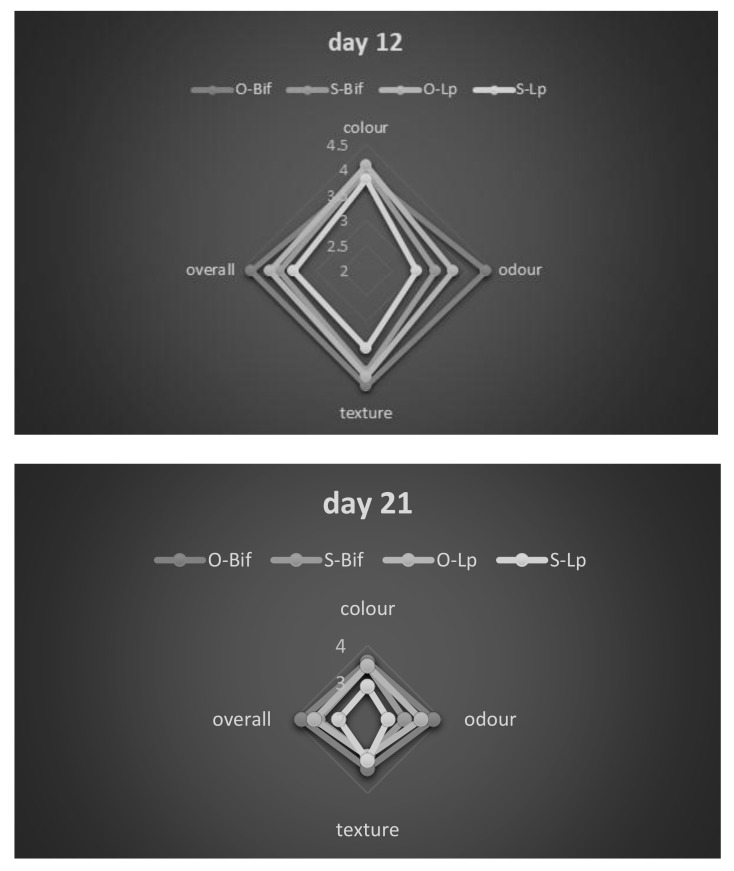
Spider graphs at days 12 and 21. O, products in sunflower oil; S, products in diluted brine; Lp, product inoculated with *L. plantarum* 69; Bif, product inoculated with *B. animalis*.

As a last step, spider profiles of samples were made at days 12 and 21 (Figure 8). These pictures offer an overview of the general sensory profile and show the worsening of the sample in the diluted brine inoculated with *L. plantarum* 69, as also suggested by MANOVA.

Some authors reported that marinated fish has a limited shelf life in refrigerated storage (e.g., 1 month), and is influenced by the quality of the raw material, the storage temperature, the levels of preservatives (salt, quantity of vinegar/acetic acid), and the possible use of other additives [24,29,30,31,32,33,34]. The shelf life of the product proposed in this research is similar, at least for the viability of the two strains. However, sensory scores were the limiting factor, and the product retained the best performance for 4 days.

In conclusion, this paper proposes a product combining functional strains and mild technologies on sea bream fillets as a prodromal step to design a new carrier for functional microorganisms. The bacteria retained a high viability throughout storage (21 days); however, the sensory scores were at their highest level for 4 days.

For the formulation, sensory assessment suggested a preference for a conditioning solution with oil, rather than with a diluted brine; in addition, a slightly higher preference was found for *B. animalis* subsp. *lactis*.

## Figures and Tables

**Table 1:** Combinations of centroid.

	Coded Values			Real Values		
	Vinegar	NaCl	Citric Acid	Vinegar (%)	NaCl (%)	Citric Acid (%)
**1**	1	0	0	70	0	**0**
**2**	0	1	0	40	12	**0**
**3**	0	0	1	40	0	**1.50**
**4**	0.50	0.50	0	55	6	**0**
**5**	0.50	0	0.50	55	0	**0.75**
**6**	0	0.50	0.50	40	6	**0.75**
**7**	0.33	0.33	0.33	50	4	**0.50**
**Control**	**-**	**-**	**-**	**-**	**-**	**-**

**Table 2:** Sensory evaluation criteria.

Quality Parameter	Description	Score
Colour	Glossy and bright surface	5
	Slight glossy and bright surface	4
	Slight glossy and dull surface	3
	No glossy appearance, 1st discoloration	2
	No glossy appearance, a little yellow surface	1
	No glossy appearance, yellow surface	0
Odour	No fishiness and no earthy smell	5
	Little fishiness, no off-odour	4
	Little freshness, no off-odour	3
	Little freshness, first off-odour	2
	Distinct freshness and off-odour	1
	Strong freshness and strong off-odour	0
Texture	Finger mark disappears immediately	5
	Finger leaves mark less than 3 s	4
	Finger leaves mark longer than 3 s	3
	Muscle returns more than half way	2
	Very soft, no elasticity	1
	Highly soft, no elasticity, no connectivity	0
Overall acceptance	Fresh, totally acceptable	5
	Little fresh, acceptable	4
	Little fresh, marginally acceptable	3
	Not fresh, unacceptable	2
	Not fresh, totally unacceptable	1
	Clearly spoiled	0

**Table 3:** Brine optimization. Critical thresholds of NaCl and citric acid for the survival of *L. plantarum* 69 and *B. animalis* subsp. *lactis* DSM 10140 (viability loss < 2 log CFU/g).

	NaCl		Citric Acid	
	Coded Values	Real Values	Coded Values	Real Values
*L. plantarum* 69	0.23	2.76%	0.63	0.95%
*L. plantarum* 68	0.04	0.50%	0.11	0.17%
*B. animalis* 10140	0.25	3%	0.50	0.75%

**Table 4:** Cell counts (log CFU/g) of *L. plantarum* 69 and *B. animalis* subsp. *lactis* DSM 10140 in fish samples (mean ± standard deviation). O, packaged in sunflower oil; S, packaged in brine.

Time (Days)	*L. plantarum* 69		*B. animalis* 10140	
	O	S	O	S
0	7.85 ± 0.20 a	7.78 ± 0.16 a	7.75 ± 0.15 a	7.80 ± 0.19 a
2	7.18 ± 0.08 d	7.20 ± 0.20 d	7.75 ± 0.24 a	7.68 ± 0.12 a
7	7.65 ± 0.18 a	7.72 ± 0.30 a	7.77 ± 0.21 a	7.34 ± 0.14 c
10	7.33 ± 0.15 c	7.50 ± 0.25 b	7.43 ± 0.19 b	7.50 ± 0.20 b
14	7.30 ± 0.22 c	7.37 ± 0.19 c	7.29 ± 0.08 c,d	7.30 ± 0.18 c
21	7.20 ± 0.10 d	7.35 ± 0.15 c	7.35 ± 0.20 c	7.45 ± 0.25 b,c

Different letters indicate significant differences (one-way ANOVA and Tukey’s test, *p* <0.05).
